# Macular Dystrophy Associated With Hereditary Spastic Paraplegia Type 11 (Kjellin Syndrome): A Multidisciplinary Case Report

**DOI:** 10.7759/cureus.106325

**Published:** 2026-04-02

**Authors:** Martina Grech, Edith Said, Rebecca Sammut, Russel Tilney, James Vassallo

**Affiliations:** 1 Ophthalmology, Mater Dei Hospital, Msida, MLT; 2 Genetics, Mater Dei Hospital, Msida, MLT; 3 Neuroscience, Mater Dei Hospital, Msida, MLT

**Keywords:** hereditary spastic paraplegia, kjellin syndrome, macular dystrophy, spastic paraplegia type 11, spg11

## Abstract

Spastic paraplegia type 11 (SPG11) is a common cause of autosomal recessive hereditary spastic paraplegia. It is frequently characterized by thinning of the corpus callosum and other neurological and extra-neurological features. Ocular involvement, including macular dystrophy, termed Kjellin syndrome, is increasingly recognized but may be clinically silent. We report a case of a 47-year-old man with an eight-year history of progressive gait disturbance and spastic paraparesis who was found to carry a likely pathogenic homozygous missense variant in the *SPG11* gene (c.5381T>C; p.Leu1794Pro). Despite minimal visual symptoms, detailed ophthalmic assessment revealed characteristic diffuse macular changes on multimodal retinal imaging. This case highlights the phenotypic spectrum of *SPG11*-associated disease and underscores the importance of ophthalmologic evaluation in patients with hereditary spastic paraplegia, even in the absence of overt visual complaints. Moreover, it highlights the varying autofluorescence properties of the observed macular lesions.

## Introduction

Hereditary spastic paraplegias (HSPs) constitute a heterogeneous group of inherited neurodegenerative disorders characterized by progressive spasticity and weakness of the lower limbs [1]. Spastic paraplegia type 11 (SPG11) represents one of the most prevalent autosomal recessive subtypes and is classically associated with thinning of the corpus callosum, cognitive impairment, and variable additional neurological features. SPG11 results from biallelic pathogenic variants in the *SPG11* gene, which encodes spatacsin, a protein widely expressed in the central nervous system and implicated in axonal maintenance [1,2].

The estimated prevalence for all types of HSP ranges from 1 to 10 per 100,000 population [2]. Since SPG11 was found to contribute 19%-31% to autosomal recessive HSP, a prevalence of around 2.5 per 100,000 population for SPG11 can be estimated [3,4].

As seen in autosomal recessive conditions, the majority of SPG11 cases emerge from countries where consanguinity is common, especially in the Mediterranean region or the Middle East [3-8]. Nevertheless, SPG11 has been reported worldwide [9,10]. Beyond the core motor phenotype, SPG11 is increasingly recognized as a multisystem disorder [2]. Less common manifestations include peripheral neuropathy, cerebellar signs, and ocular abnormalities.

Retinal degeneration associated with SPG11, also known as Kjellin syndrome, is a rare condition characterized by macular changes resembling, but distinct from Stargardt’s disease. Kjellin syndrome is characterized based on the retinal appearance in a patient with spastic paraplegia and thin corpus callosum. Importantly, retinal involvement may be asymptomatic, remaining undiagnosed unless active screening is performed.

Here, we present a detailed clinical, genetic, and ophthalmic description of a middle-aged man with SPG11-associated hereditary spastic paraplegia and characteristic macular changes, including autofluorescence images. 

## Case presentation

A 47-year-old man was referred for neurological assessment in September 2023 in view of an eight-year history of gait disturbance. He dragged his legs on walking and reported nocturnal calf cramping. He also complained of hearing loss and mild urinary urgency, but denied other sensory symptoms and urinary incontinence. A mild, gradual worsening near vision was reported. The rest of the history was unremarkable and there was no known family history.

**Figure 1:**
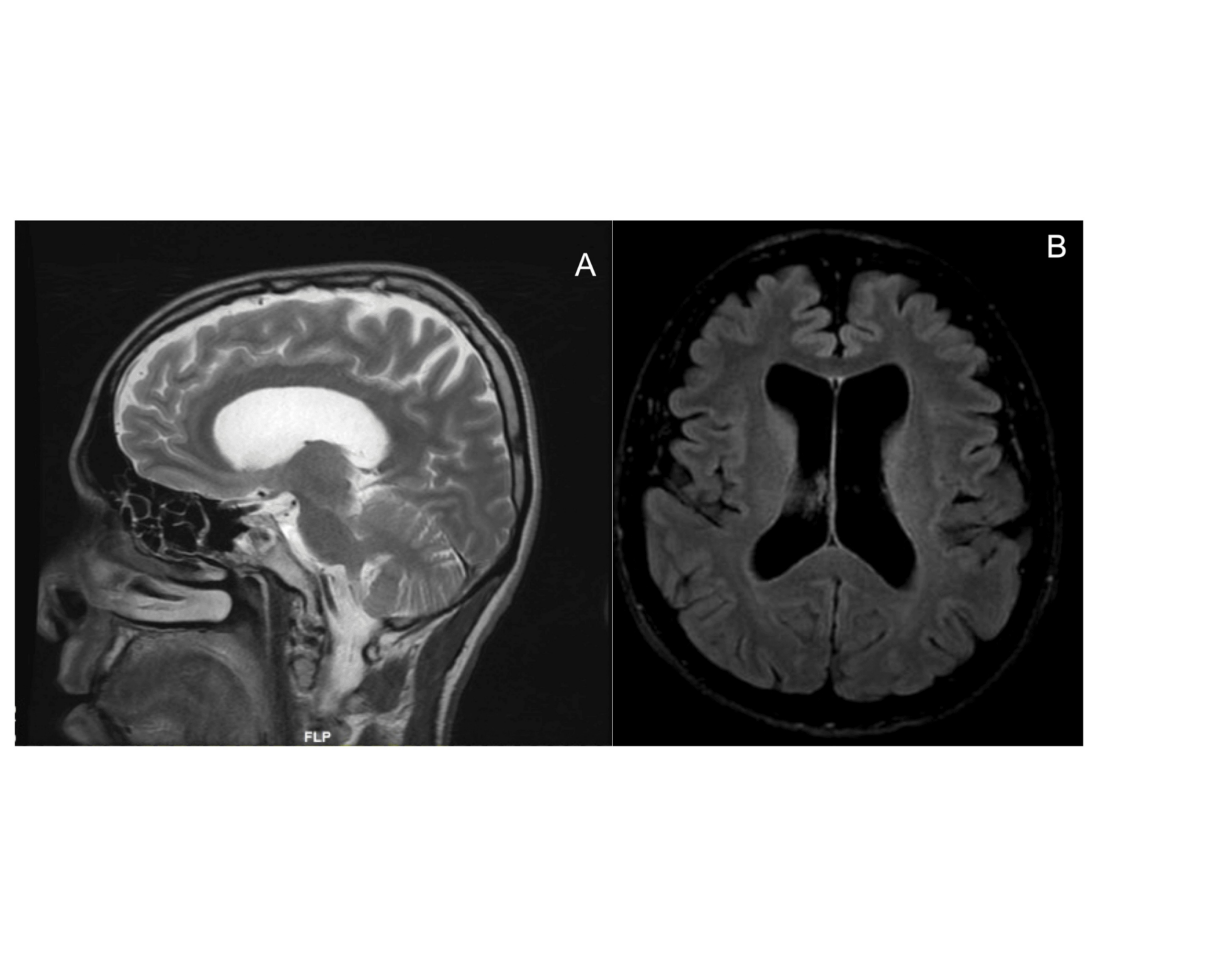
MR head: Sagittal T2 (A) and axial T2 FLAIR (B) showing isolated mild cerebral atrophy FLAIR: Fluid-attenuated inversion recovery.

On examination, spasticity of the lower limbs was elicited, with bilateral hyperreflexia of the knee and ankle jerks. Bilateral suprapatellar reflexes were elicited and Babinski reflexes were upgoing. A spastic gait was observed. Serum aldolase was elevated, but creatinine kinase levels were normal. Autoimmune and infective screens were negative. Serum alpha- and beta-glucosidase, copper and homocysteine levels were normal. Electromyography showed myopathic features. Lumbar puncture was normal. Magnetic resonance (MR) brain imaging showed mild cerebral atrophy only (Figure 1). Echocardiography was normal and no arrhythmias were detected on Holter monitoring. Urological evaluation was unremarkable. Oral baclofen was initiated and titrated gradually to alleviate spasticity.

The patient was tested by next-generation sequencing of 41Mb of the human coding exome with at least 20x coverage depth for >98% of the targeted bases (Centogene GmbH, Rostock, Germany). A homozygous missense variant, c.5381T>C p.(Leu1794Pro), was identified in the SPG11 gene. It is classified as likely pathogenic (class 2) according to the recommendations of CENTOGENE (CENTOGENE GmbH, Rostock, Germany) and American College of Medical Genetics and Genomics (ACMG) [11]. No parent consanguinity was reported. Moreover, parental targeted testing and familial cascade carrier testing were recommended for the patient, as he has a son. 

The patient was referred to ophthalmology in view of presbyopia. Given the known associations between SPG11 and retinal involvement, and the gradual worsening of near vision, a referral to ophthalmology was done by neurology on first presentation. Initial ophthalmic assessment was done in September 2024, where he denied any visual symptoms apart from presbyopia. Visual acuity was 20/20+ in both eyes. Anterior segment examination and intraocular pressures were within normal limits. Fundoscopy showed bilateral scattered drusen-like changes in the macula with foveal pigmentary changes; the right fovea appeared darker, while the left fovea was hypopigmented. The discs and fundal peripheries were normal. 

**Figure 2:**
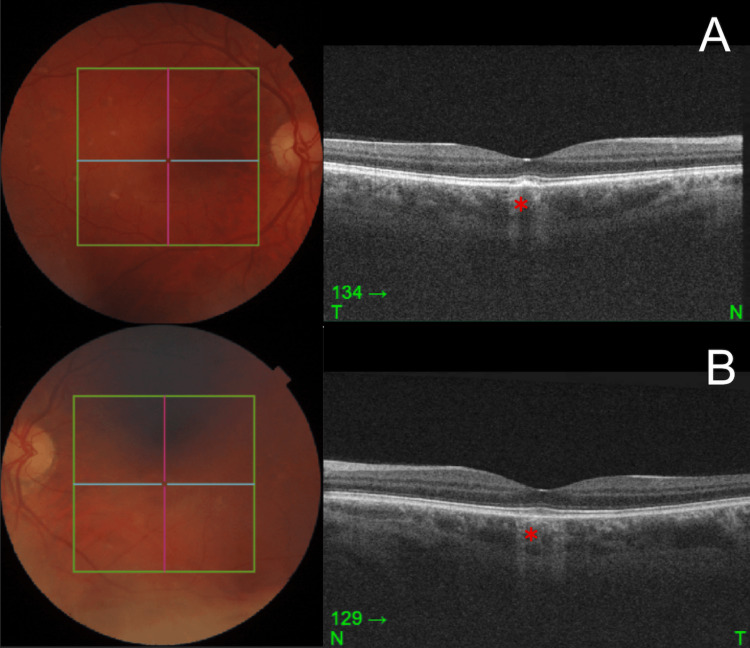
Macular OCT using Topcon DRI OCT Triton (red asterisk: The right fovea showed localized photoreceptor hyperreflectivity, and there was bilateral disruption of the interdigitation zone and increased signal transmission; A: right macula OCT; B: left macula OCT) OCT: Optical coherence tomography

Humphrey central automated perimetry was normal on the right, but showed a mild superior extension of the left blind spot. Goldmann kinetic perimetry was normal. Optical coherence tomography (OCT), done in March 2025, revealed that the right fovea showed localized photoreceptor hyperreflectivity, and there was bilateral disruption of the interdigitation zone and increased signal transmission (Figure 2). Increased signal transmission indicates retinal pigment epithelial attenuation.

**Figure 3:**
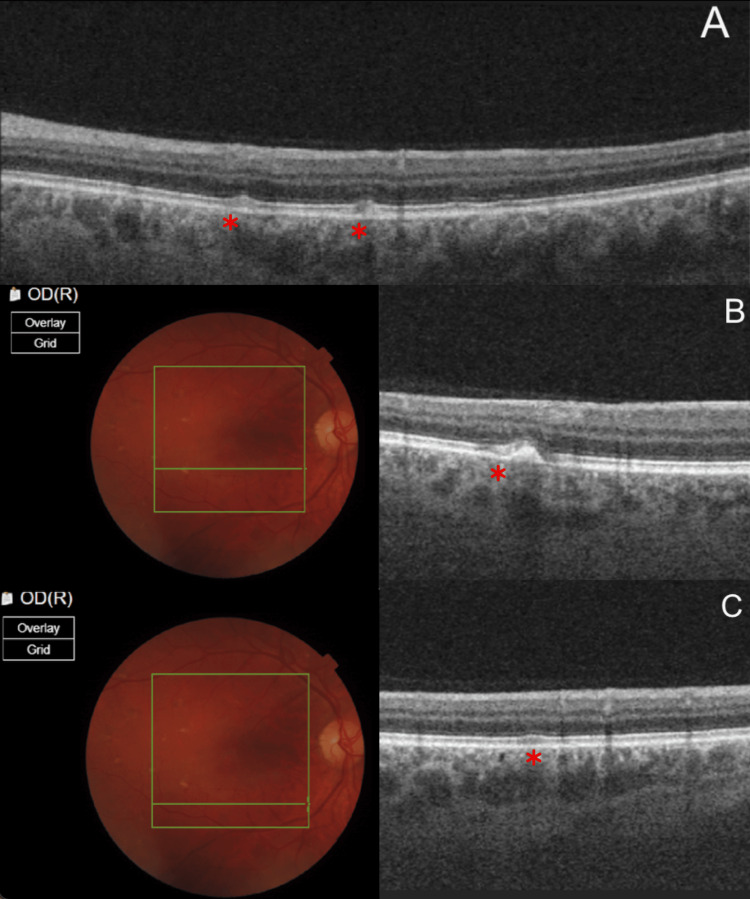
(A) Section through the drusen-like lesions (marked by red asterisk) showed small-medium sized, moderately reflective mounds in the photoreceptor layer with disruption of the interdigitation zone and variable attenuation and upwards deviation of the ellipsoid zone, with protrusion towards and inner deviation of the external limiting membrane. The lesions were in different stages of severity (B) A section through a hyperautofluorescent lesion revealed the drusen-like lesion (marked by red asterisk) (C) A section through a hypoautofluorescent lesion was unremarkable (marked by red asterisk)

**Figure 4:**
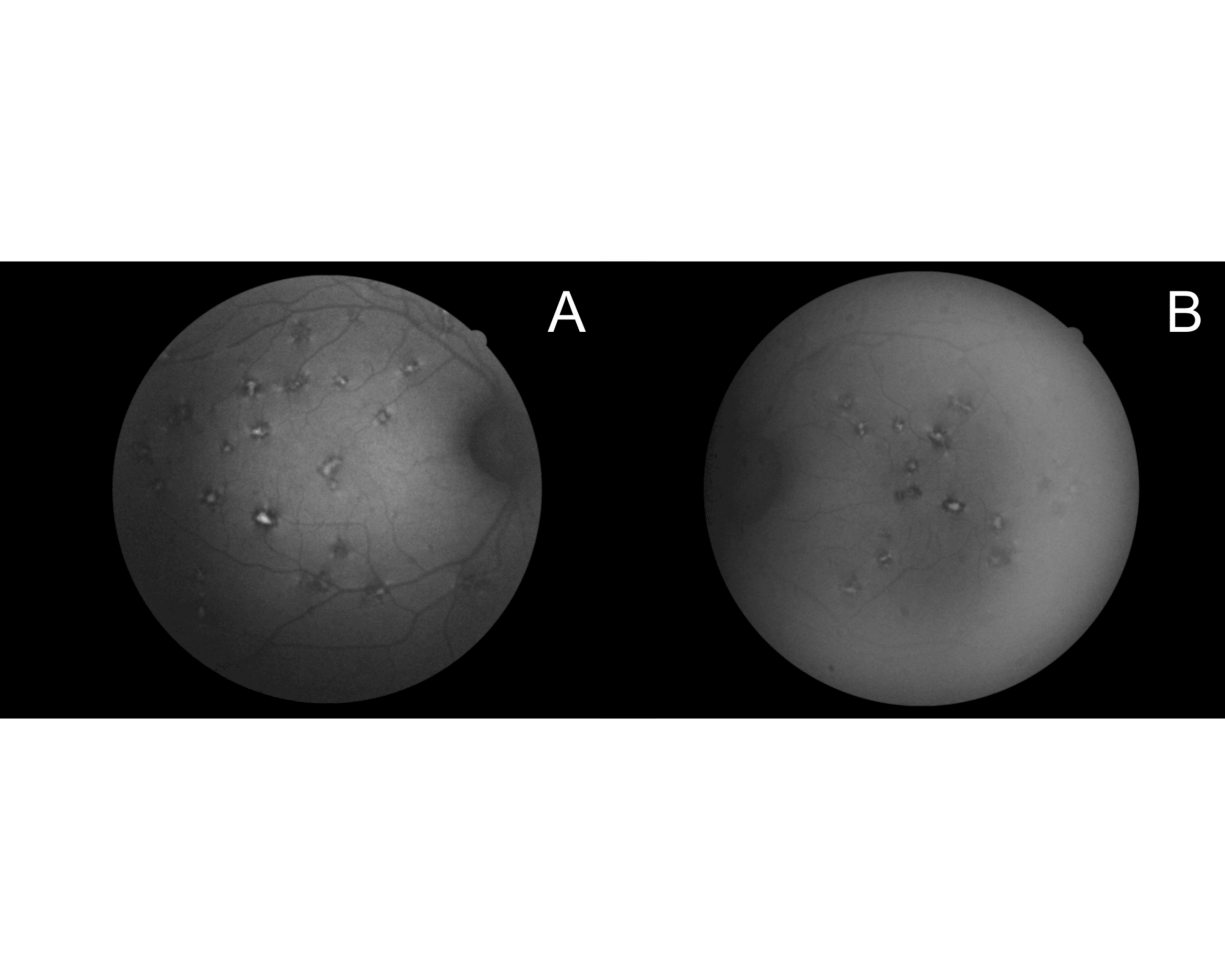
Fundus autofluorescence photos of the right and left posterior poles revealing a reticular pattern of discrete amoeboid hyperautofluorescent lesions with a thick hypofluorescent rim (A: right posterior pole; B: left posterior pole)

The OCT appearance of the macular drusen-like lesions varied according to their autofluorescence (Figures 3, 4). This implies that lesions evolve over time with eventual resorption, and new lesions (likely hyperautofluorescent) coexisting with older ones (likely hypoautofluorescent). Blue autofluorescence photos of the posterior poles revealed a reticular pattern of discrete amoeboid hyperautofluorescent lesions with a thick hypofluorescent rim. Some lesions, especially those close to the main arcades, were mostly hypoautofluorescent.

From an ophthalmology point of view, the patient is currently being regularly followed up with OCT and fundus autofluorescence (FAF) imaging to monitor for progression. Moreover, he is being followed up by neurology every six months. Baclofen dose was increased to 10 mg three times daily during his last follow-up in view of increased spasticity, and the patient was also referred for physiotherapy. 

## Discussion

Hereditary spastic paraplegias are characterized by gradually increasing symptoms and signs [1]. Patients may also present with paresthesia, or pain in the arms and legs due to sensory nerve involvement, motor neuropathy, intellectual disability, hyperreflexia of the lower limbs, dysarthria, decreased bladder control and loss of muscle mass. Less frequently, individuals may experience dysphagia, pes cavus, abnormal curvature of the spine, nystagmus, and retinal degeneration. Onset occurs mainly during infancy or adolescence, but in rare cases it starts at around 60 years.

In this case, mild cerebral atrophy was the only neuroimaging feature noted. Other radiological findings may include spinal cord atrophy, corpus callosum atrophy, increased T2 signal intensity in the posterior limb of the internal capsule, ears of the lynx sign (high T2/fluid-attenuated inversion recovery (FLAIR) signal intensity at the tips of the frontal horns of the lateral ventricles), hypointense T2 signal of the globi pallidi, hydrocephalus and cerebellar atrophy [12]. Thinning of the corpus callosum, seen in >90% of individuals, with long T1 and T2 values in the forceps minor of the corpus callosum, referred to as "ear of the lynx" sign, which appears hyperintense on FLAIR and hypointense on T1-weighted images, is usually present [2,3,13]. In a large case series, 60% of individuals with thinning of the corpus callosum, cognitive impairment, and spastic paraparesis were identified as having biallelic SPG11 pathogenic variants [3].

Ocular manifestations may include: macular excavation or degeneration as described in Kjellin syndrome; strabismus; cerebellar signs, including abnormal saccadic pursuit and nystagmus; and prolonged latencies and reduced amplitudes on visually evoked potentials [2,14,15]. Kjellin syndrome classically describes the association of retinal degeneration, autosomal recessive hereditary spastic paraplegia, and a thin corpus callosum [2]. It was first described in association with spastic paraplegia type 15 (SPG15) but is more common with SPG11. Ophthalmology evaluation is advisable in patients with comparable neurologic deficits, as it is critical for diagnosis, and visual symptoms may be absent despite clinically apparent retinal involvement as seen in our case.

Macular abnormalities in Kjellin syndrome resemble those seen in Stargardt’s disease, but they differ in the appearance, distribution, angiographic features, and autofluorescence patterns of the retinal flecks [16]. The pathogenic gene product in Kjellin syndrome appears to lead to progressive dysfunction across multiple neuronal tissues and is distinct from the primary defect responsible for the Stargardt disease phenotype. Stargardt disease (STGD1) is an autosomal recessive maculopathy characterized by lipofuscin accumulation, central vision loss, and peripapillary sparing, often with an early onset. SPG11-related spastic paraplegia, a neurodegenerative disorder, presents with a similar Stargardt-like macular degeneration but is associated with thin corpus callosum, cognitive impairment, and a slower progression.

Moreover, Kjellin syndrome presents with retinal flecks that phenotypically resemble age-related macular degeneration (AMD) but are pathologically distinct, representing an early-onset, metabolic lysosomal disorder rather than age-related lipid accumulation. 

SPG11 is diagnosed in a proband based on characteristic MRI findings and clinical features, along with the identification of pathogenic variants in both copies of the SPG11 gene through molecular genetic testing [2]. Spastic paraplegia type 11 is caused by missense mutations in the SPG11 gene, which encodes a protein known as spatacsin. The variant found in this case has been previously described as disease-causing [2,17-19]. Parents usually don’t exhibit any features of the condition.

Spatascin is expressed throughout the nervous system, although its precise function is unknown. It may play a role in axon maintenance [1]. The expression of spatacsin has been demonstrated in cortical and spinal motor neurons in particular, as well as in the retina [20]. SPG11 mutations alter the structure of the spatacsin protein, thus possibly leading to the neurological features.

Our variant was highly conserved and predicted to be probably damaging by PolyPhen and Mutation Taster and was determined to be likely pathogenic in accordance with the American College of Medical Genetics and Genomics (ACMG) guidelines [11].

The management of this condition is best provided through a multidisciplinary team. Physiotherapy is done to mobilize spastic muscles. Antispastic medications, for example oral baclofen, may be prescribed, while botulinum toxin injections or intrathecal baclofen can be considered for severe, disabling spasticity when oral treatments are ineffective [2]. Psychiatric symptoms should be treated according to standard clinical guidelines. Urodynamic studies are recommended when bladder dysfunction is present, with anticholinergic medications used to manage urinary urgency. Clinical evaluations are recommended every six months to readjust physiotherapy regimens and medications as needed.

Moreover, genetic counselling is important in such cases. Genetic testing for at-risk family members, including prenatal, is possible once the pathogenic variants in a family are identified.

## Conclusions

This case illustrates the value of multidisciplinary care and contributes to the growing body of literature defining the ocular phenotype of SPG11. SPG11 is a complex neurodegenerative disorder with extra-neurological manifestations, including retinal involvement. Despite preserved visual acuity and minimal subjective symptoms, multimodal ophthalmic imaging can reveal characteristic macular abnormalities. Recognition of these features is important for accurate phenotyping, diagnosis, and longitudinal monitoring. Hence, ophthalmologic screening in individuals with SPG11 and related hereditary spastic paraplegias is recommended, particularly given the potential diagnostic value of retinal changes and their role in differentiating SPG11 from other neurogenetic and retinal disorders. Early identification also facilitates appropriate counselling, multidisciplinary management, and improved understanding of the full phenotypic spectrum associated with SPG11 mutations.
